# Disrupted gray matter structural covariance networks in chronic insomnia disorder

**DOI:** 10.3389/fpsyt.2025.1629534

**Published:** 2026-01-07

**Authors:** Zhonglin Li, Yu Shen, Jiao Liu, Zhi Zou, Xiaoling Wu, Yuang Gu, Hui Gao, Miao Zhang, Ao Liu, Qi Qiao, Shulei Jia, Xinbei Lin, Yawei Du, Yang Zhou, Yongbing Sun, Ling Wang, Fengshan Yan, Shewei Dou, Hao Li, Li Tong, Xue Lv, Yongli Li

**Affiliations:** 1 Department of Radiology, Henan Provincial People’s Hospital & People’s Hospital of Zhengzhou University, Zhengzhou, China; 2 Henan Key Laboratory of Imaging and Intelligent Processing, Information Engineering University, Zhengzhou, China; 3 Department of Nuclear Medicine, The First Affiliated Hospital of Zhengzhou University, Zhengzhou, China; 4 Department of Nuclear Medicine, Henan Provincial People’s Hospital & People’s Hospital of Zhengzhou University, Zhengzhou, China; 5 Department of Radiology, Xinxiang Medical University, Henan Provincial People’s Hospital, Zhengzhou, China; 6 Department of Radiology, Henan University People’s Hospital, Henan Provincial People’s Hospital, Zhengzhou, China; 7 Department of Health Management, Fuwai Central China Cardiovascular Hospital, Zhengzhou, China; 8 Health Management Center, Henan Provincial People’s Hospital & People’s Hospital of Zhengzhou University, Zhengzhou, China

**Keywords:** chronic insomnia disorder, gray matter volume, graph theory, structural covariant network, topological property, betweenness centrality

## Abstract

**Background:**

Chronic insomnia disorder (CID) is associated with changes in gray matter volume (GMV) and structural connectivity in several brain regions. However, alterations in the topological properties of the structural covariant network (SCN) remain poorly understood in CID.

**Methods:**

Voxel-based morphometry and graph theory were applied to examine the topological characteristics of the GMV-based SCN in 82 patients with CID and 73 healthy controls. Group comparison of GMV and multiple regression with pittsburgh sleep quality index (PSQI) were conducted, with hamilton depression acale, hamilton depression scale, total intracranial volume, age, sex, and years of education as covariates. The brain SCN was constructed by thresholding Pearson correlations between the corrected GMVs of 90 brain regions, defined via the automated anatomical labeling atlas. Both the global and nodal topological properties of the brain SCN were analyzed, controlling for the same set of covariates.

**Results:**

The bilateral precentral gyrus (PreCG) showed both increased GMV and a negative correlation with PSQI scores (*p* < 0.001, uncorrected). No significant differences were found in the global network topological properties between groups. CID patients exhibited increased nodal betweenness centrality in the right paracentral lobule (PCL), and decreased nodal degree and efficiency in the left postcentral gyrus (PoCG) (*p* < 0.05, false discovery rate corrected). Furthermore, we observed alterations in both the number and distribution of network hubs. Notably, the constellation of regions exhibiting altered nodal parameters (the right PCL and left PoCG) also functioned as reconfigured network hubs.

**Conclusions:**

This study establishes an association between sleep disturbances in CID and aberrations in both the GMV of specific sensory-motor network nodes (PreCG, PCL, PoCG) and their SCN topological properties, thereby providing new directions for elucidating the disorder’s pathophysiology.

## 1 Introduction

Chronic insomnia disorder (CID) is a significant global public health issue, a concern further amplified by the COVID-19 pandemic (1, 2). CID is characterized by persistent difficulties in falling asleep, frequent nighttime awakenings, and early morning awakenings (1). These symptoms not only diminish daily quality of life and impair work efficiency but also contribute to serious mental health conditions, thereby elevating the risk of life-threatening outcomes (1, 3). Consequently, identifying the neural alterations underlying CID is critical for elucidating the mechanisms of sleep disturbances and for developing more effective diagnostic and therapeutic strategies.

Advanced structural magnetic resonance imaging (MRI) techniques provide insights into brain alterations beyond the capabilities of conventional MRI, making them powerful tools for investigating CID (4, 5). Voxel-based morphometry (VBM) studies have linked CID to gray matter volume (GMV) alterations in regions including the frontal, temporal, and parietal cortices (4–11). However, these findings are inconsistent. For instance, Yu et al. reported significantly increased cortical volume in the left orbital frontal cortex (OFC), right rostral anterior cingulate cortex, and right fusiform gyrus (FFG) in primary insomnia patients compared to healthy controls (HCs) (8). Similarly, Li et al. observed GM hypertrophy in the left anterior/middle cingulate gyrus and right middle/inferior temporal gyrus in CID patients (9). In contrast, Joo et al. found GM reductions in the bilateral frontal lobes, among other regions, in patients with psychophysiologic insomnia (10), while Altena et al. reported reduced GMV in the OFC that correlated with subjective insomnia severity (11). These disparate findings suggest that a focus on isolated brain regions may be insufficient. The brain functions as a complex information-processing system reliant on the coordinated activity of distributed networks (6, 12, 13). Notably, the altered cognitive performance, emotional processing, and memory formation observed in CID patients are associated with disruptions across widely distributed brain regions and subnetworks (6, 12, 13). Therefore, investigating the neural mechanisms of CID from a structural network perspective is essential.

Recent neuroimaging studies propose the structural covariance network (SCN) as a valuable tool for investigating brain topology (14–17). The SCN characterizes the topological organization of brain structures, revealing hierarchical brain architecture, intrinsic cortical organization, and co-varying changes across regional measures (14–17). This capacity makes it particularly suitable for describing the brain’s intrinsic properties throughout development and aging, as well as the impact of environmental factors and chronic disease (14–17). Furthermore, compared to time series-based functional networks and diffusion tensor imaging-based anatomical networks, GMV-based SCN analysis offers the advantages of relatively low computational complexity and greater robustness to noise (18, 19).

Several seed-based SCN studies have investigated structural network disruptions in CID. Zhao et al., focusing on sensory regions, reported increased structural covariance in cortical thickness between sensory and motor areas in patients (20). In a similar analysis of the default mode network (DMN), Suh et al. found that the disrupted SCN in CID patients might reflect a malfunction in the antero-posterior disconnection of the DMN during the wake-to-sleep transition (21). Elsewhere, Chou et al. applied a seed-based GMV SCN analysis to patients with comorbid migraine and insomnia, identifying decreased structural covariance integrity in the cerebellum (22). Although these studies have successfully identified altered structural connectivity between specific brain regions using GMV or cortical thickness, the overarching changes in the global topological properties of the SCN in CID patients remain elusive.

Graph theory provides a powerful framework for quantitatively characterizing the topological organization of large-scale brain networks at both global and nodal levels (23, 24). In our previous work, we applied this approach to reveal disruptions in functional network topology in CID, observing a reduced number of modules, simplified hierarchies, and increased assortativity (25). Separately, Yang et al. employed graph-theoretical analysis on a GMV-based SCN in patients with temporal lobe epilepsy and comorbid sleep disorder, reporting significantly increased clustering coefficients, shortest path length, transitivity, and local efficiency (4). Despite these insights, the topological properties of the GMV-based SCN in patients with CID remain unknown.

Building on prior evidence of disrupted functional network topology and regional GMV alterations in CID, we hypothesized that CID would be associated with abnormal topological organization in the GMV-based SCN. To test this, we employed graph theory to analyze key topological properties, including small-worldness, global/local efficiency, nodal degree, nodal efficiency, and nodal betweenness centrality (BC). Our analysis proceeded in three stages: first, we used VBM to identify GMV differences between CID patients and HCs; second, we examined correlations between GMV and clinical variables; finally, we compared the global and nodal topological properties of the GMV-based SCN between the two groups.

## 2 Materials and methods

### 2.1 Participants

The study design and ethical approval for this study were obtained from the Ethics Committee of Henan Provincial People’s Hospital (approval number: [2021(67)]). All patients provided informed consent and received compensation for their participation. Specialized and experienced neurologists conducted sleep-related interviews and administered a standardized screening to exclude other sleep disorders or comorbid conditions. All participants underwent a comprehensive neuropsychological and clinical assessment, including the Pittsburgh Sleep Quality Index (PSQI) (26), the 17-item Hamilton Depression Scale (HAMD) (27), and the 14-item Hamilton Anxiety Scale (HAMA) (28). Participants were included based on the criteria outlined in the Fifth Edition of the Diagnostic and Statistical Manual of Mental Disorders, as follows: (1) the presence of fatigue, irritability, cognitive decline, or other insomnia symptoms lasting for at least 3 months; (2) a PSQI score ≥ 8 according to the latest study by Zhang et al. (29); (3) no history of psychiatric or neurological disorders (e.g., schizophrenia, stroke); (4) no secondary sleep disorders (e.g., restless legs syndrome, obstructive sleep apnea); (5) age between 18 and 70 years; (6) right-hand dominance (determined by the Chinese handedness inventory designed for Chinese people, which includes 10 test items) and being a native Chinese speaker; (7) no history of alcohol or substance abuse or dependence; and (8) no brain lesions or prior significant head trauma, as confirmed by T2-weighted dark-fluid and T1-weighted MR images. Given the potential associations between CID and both depression and anxiety (30, 31), and based on the latest study (32), we did not limit the depression and anxiety scores during the recruitment of patients with CID. HCs were required to meet the following criteria: (1) no history of sleep disorders (PSQI ≤ 7); (2) good sleep quality and no history of shift work; and (3) fulfillment of the inclusion criteria 3 to 8 listed for CID patients. A total of 155 participants were recruited: 82 patients with CID and 73 HCs. The groups were matched for sex, age, and years of education (**Table 1**).

**Table 1:** Demographics and clinical characteristics of the participants.

Variables	CID (n=82)	HCs (n=73)	*P* value
Age (years)	37.5 (22.3)	37.4 ± 10.0	0.100
Gender (male/female)	26/56	25/48	0.737^a^
Education (years)	15.0 (4.0)	15.0 (2.0)	0.294
Total Intracranial Volume	1434.9 ± 112.8	1459.1 (138.8)	0.243
Gray Matter Volume	660.6 ± 52.6	674.7 ± 54.3	0.267
White Matter Volume	519.9 ± 45.1	521.2 ± 51.9	0.113
Cerebrospinal Fluid Volume	246.2 (55.1)	259.6 ± 43.7	0.175
Insomnia Duration (years)	5.0 (8.0)	N/A	N/A
PSQI	15.0 (3.0)	2.0 (2.0)	<0.001
HAMA	13.0 (10.5)	1.0 (2.5)	<0.001
HAMD	11.5 (10.0)	0.0 (2.0)	<0.001

Normally distributed data are expressed as mean ± SD. Non-normally distributed data are presented as median and inter-quartile range, and p values were obtained by Wilcoxon signed-rank test. The ^a^*p* value was obtained by two-tailed Pearson chi-square test. Abbreviation: CID, chronic insomnia disorder; HCs, healthy controls; PSQI, Pittsburgh Sleep Quality Index; HAMA, Hamilton Anxiety Rating Scale; HAMD, Hamilton Depression Rating Scale.

### 2.2 Data acquisition

MRI data acquisition was performed at the medical imaging center of our hospital via MAGNETOM Prisma 3T MR scanner (Siemens Healthcare, Erlangen, Germany) equipped with a 64-channel head–neck coil. To ensure participant comfort and minimize motion artifacts, earplugs and foam pads were provided to reduce scanner noise and limit head movement. Additionally, non-allergenic tape was gently applied to the participants’ foreheads as a tactile reminder to minimize movement. High-resolution T1-weighted structural images were obtained with the following parameters: repetition time: 2,300 ms, echo time: 2.27 ms, field of view: 250 mm × 250 mm, matrix size: 256 × 256, 192 slices with a slice thickness of 1 mm, and a flip angle of 8°. Prior to further image processing, all images were reoriented to set the anterior commissure as the origin using a center-of-mass approach.

### 2.3 GMV analysis

Voxelwise GMV maps were derived from the T1-weighted images using a VBM approach (33). The Computational Anatomy Toolbox (CAT12) (33) within the Statistical Parametric Mapping software (SPM12; Wellcome Trust Centre for Neuroimaging), implemented in MATLAB R2018a (Mathworks, Sherborn, MA, USA), was employed to extract both subcortical and cortical regional GMV. The data processing involved the following steps: (1) spatial normalization of the images to the Montreal Neurological Institute space using the ICBM152 template; (2) segmentation into gray matter (GM), white matter, and cerebrospinal fluid (CSF); (3) modulation of the gray matter segments to preserve the total amount of gray matter; and (4) spatial smoothing of the modulated and normalized gray matter maps using an 8-mm full-width-at-half-maximum Gaussian kernel. Scans with an overall image quality rating below B+ were excluded from subsequent analyses. Following these steps, the data quality of the modulated GM segments was assessed using the CAT12 “Check Sample Homogeneity” function along with careful visual inspection. No participants were identified as outliers. Whole-brain voxelwise group differences in GMV were assessed using a general linear model, with HAMA score, HAMD score, total intracranial volume (TIV), age, sex, and years of education included as covariates.

### 2.4 SCN analysis

The average GMV of 90 cortical and subcortical regions of interest (ROIs) from the Automated Anatomical Labeling atlas was calculated using the CAT12 toolbox (25, 34). A linear regression analysis was performed for each ROI to account for the effects of HAMA score, HAMD score, TIV, age, sex, and years of education on GMV. For each group, a 90 × 90 adjacency matrix was created based on Pearson correlation coefficients between the corrected GMV. Only positive correlations were retained as edges (i.e., connections), and negative correlations were set to zero for the subsequent network analysis. The correlation matrices were then binarized with a fixed sparsity threshold to ensure that both groups had the same number of edges in the binarized network. A wide range of sparsity thresholds (0.10 to 0.50, in steps of 0.02) was used, following previous literature (35–37). The lower bound of this range was defined as the minimum density at which the networks for both groups remained connected (i.e., contained no isolated components; 0.10 in this study). Thresholds above 0.50 were excluded to avoid networks that become increasingly random and lose their small-world characteristics (i.e., small-world indices approach 1). The Brain Connectivity Toolbox (38) was employed to estimate the topological properties of the SCN. Both global and regional topological properties were estimated. Global network parameters included the small-world index, clustering coefficient, path length, global efficiency, and local efficiency. Regional (nodal) properties included nodal degree, nodal efficiency, and nodal BC.

### 2.5 Network hub analysis

Network hubs are nodes that play a crucial role in facilitating information flow. In this study, we normalized the nodal BC values. Following previous studies (34, 39), a node was defined as a hub if its normalized BC exceeded the network’s average by at least one standard deviation.

### 2.6 Statistical analysis

#### 2.6.1 Demographic and clinical data analysis

All statistical analyses were performed using Statistical Product and Service Solutions version 26.0 (SPSS 26.0; Chicago, IL). The distributions of continuous variables, including age, years of education, duration of insomnia, PSQI, HAMA, HAMD, and TIV, were tested using the Kolmogorov–Smirnov test. Continuous variables with a normal distribution were analyzed using independent two-sample t tests and are presented as means ± standard deviations. Non-normally distributed data were analyzed using the Mann–Whitney U test and are reported as medians and interquartile ranges. Gender and education differences between the two groups were assessed using a Pearson chi-square test. The threshold for statistical significance was set at *p* < 0.05, and all hypothesis tests were two-tailed.

#### 2.6.2 Group comparison of GMV and multiple regression

Statistical and multiple regression analyses of GMV were conducted using Cat12/SPM12. A voxelwise two-sample t-test was used to examine differences in the GMV between patients with CID and HCs, controlling for HAMA scores, HAMD scores, TIV, age, sex, and years of education. Furthermore, a whole-brain multiple regression analysis was conducted to investigate the correlation between GMV and PSQI scores in the CID group, controlling for the same covariates (10, 40). Family wise error correction was performed for multiple comparison correction. However, no significant differences were found between the two groups after FWE correction. Therefore, a less stringent statistical threshold was employed, using an uncorrected voxelwise threshold of *p* < 0.001 with a cluster threshold of 26 voxels, an approach that has been applied in previous studies on structural differences in patients with CID (10, 41). To contextualize the findings obtained with this uncorrected threshold and aid in their interpretation, we calculated and reported Cohen’s d to quantify the effect size of the between-group differences.

#### 2.6.3 Group comparison of global and regional topological properties

Non-parametric permutation testing (1000 repetitions) was conducted to test for CID-related differences in global and regional network parameters, controlling for the HAMA score, HAMD score, TIV, age, sex, and years of education (32, 35, 36). For each thresholded network, the topological properties of the SCN were estimated. For each permutation, group labels were randomly shuffled, and the differences between the newly formed groups were computed to build a permutation distribution under the null hypothesis. To summarize the overall group-level differences across the range of network densities, the area under the curve (AUC) for each topological metric was computed (32, 35, 36). A significance level of *p* < 0.05 was used for all tests, with statistical significance for group differences in global and regional topological properties determined after false discovery rate (FDR) correction for multiple comparisons.

## 3 Results

### 3.1 Demographic and clinical data

**Figure 1:**
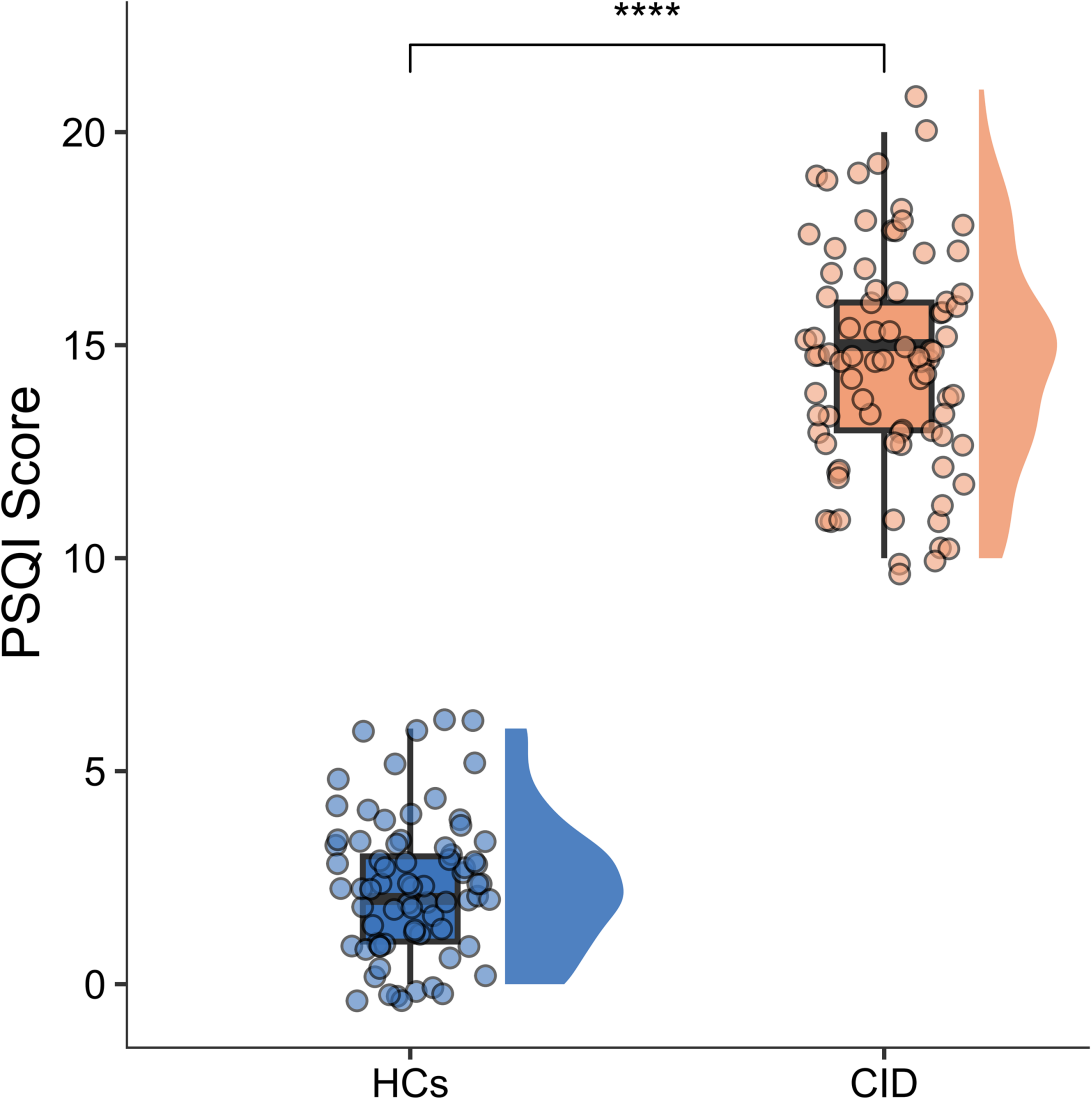
Dot plots of pittsburgh sleep quality index scores for the HCs and CID groups. HCs, healthy controls; CID, chronic insomnia disorder. **** indicates p < 0.0001.

In this study, a total of 82 patients with CID and 73 HCs were included for further analysis. The demographic data for the patients with CID were as follows: 56 females; education: 15.0 (4.0) years; and age: 37.5 (22.3) years. The demographic data for the HCs were as follows: 48 females; education: 15.0 (2.0) years; and age: 37.4 ± 10.0 years. Among the clinical data, age, GMV, and white matter volume (WMV) in HCs, as well as TIV, GMV, WMV, and CSF volume in CID patients, were normally distributed. All other demographic and clinical data deviated from a normal distribution. No significant differences were found in age, sex, years of education, TIV, GMV, WMV, or CSF volume between patients with CID and HCs (*p* > 0.05; **Table 1**). In contrast, significant between-group differences were observed in the PSQI, HAMA, and HAMD scores (*p* < 0.05; **Table 1**). **Figure 1** displays dot plots of PSQI scores for the CID and HCs groups.

### 3.2 GMV differences and multiple regression results

**Figure 2:**
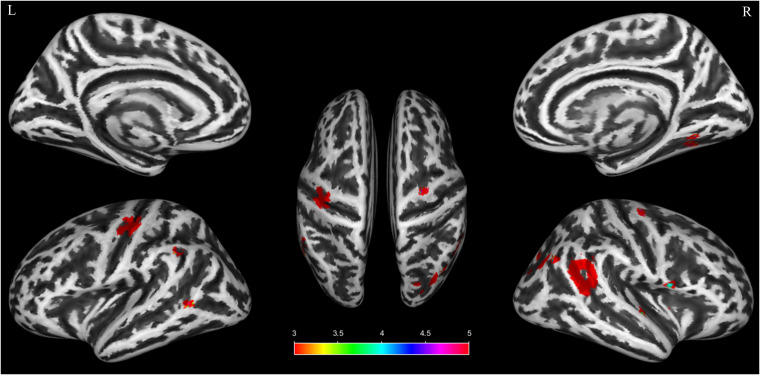
Brain areas that exhibited altered GMV in patients with chronic insomnia disorder compared with healthy controls. The results were set at the voxel level: *p* < 0.001, cluster ≥ 26 voxels. Warm colors indicate regions in which the GMV markedly increased. The color bar indicates the *t* value. GMV, gray matter volume; L, left; R, right.

Compared with HCs, patients with CID had larger GMV in several brain regions, including the bilateral precentral gyrus (PreCG), as well as the right insula, right temporal pole-superior temporal gyrus (TPOsup), left middle temporal gyrus (MTG), right superior temporal gyrus (STG), right FFG, right angular gyrus (ANG), left inferior parietal gyrus (IPG), left postcentral gyrus (PoCG), and right middle occipital gyrus (MOG). The spatial distribution of these regions is depicted in **Figure 2**. The results were thresholded at the voxel level at *p* < 0.001, with a cluster extent of 26 voxels, uncorrected. **Table 2** provides detailed information on these significant GMV clusters. To quantify the effect sizes, we have added Cohen’s d to **Table 2**.

**Table 2:** Brain regions exhibited altered gray matter volume in patients with chronic insomnia disorder when compared with healthy controls.

Brain regions	Anatomical classification	Whole cluster size	Region size	MNI coordinates			t score	Cohen’s d
				x	y	z		
R Insula	Subcortical	131	41	48	0	0	3.66	0.60
R Temporal pole: superior temporal gyrus	Temporal		10	62	6	2	3.49	0.58
L Middle temporal gyrus	Temporal	27	20	-62	-53	3	3.61	0.60
R Superior temporal gyrus	Temporal	505	318	57	-53	21	4.86	0.80
R Fusiform gyrus	Temporal	42	35	30	-51	-9	3.48	0.57
R Angular gyrus	Parietal	38	30	44	-71	32	3.82	0.63
L Inferior parietal gyrus	Parietal	26	20	-56	-50	38	4.19	0.69
L Postcentral gyrus	Parietal	328	249	-38	-20	48	4.86	0.80
L Precentral gyrus	Frontal		79	-35	-29	54	3.66	0.60
R Precentral gyrus	Frontal	82	72	30	-15	69	4.25	0.70
R Middle occipital gyrus	Occipital	211	205	35	-83	32	3.78	0.62

Results were set at *p* < 0.001 (uncorrected, cluster ≥ 26 voxels). Abbreviations: MNI, Montreal Neurological Institute; L, left; R, right.

**Figure 3:**
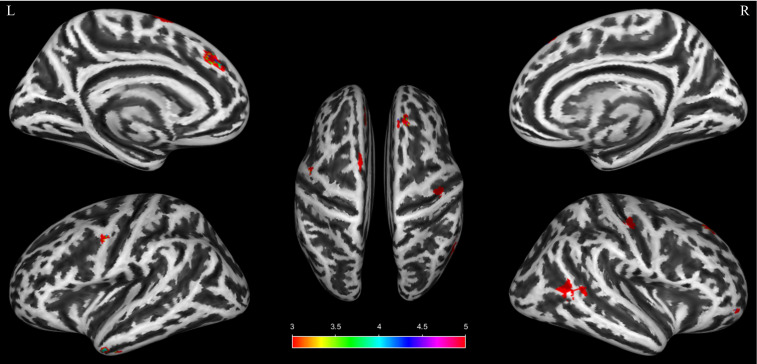
Multiple regression analysis revealed a significant negative correlation between gray matter volume and pittsburgh sleep quality index scores in several brain regions among participants with chronic insomnia disorder. Warm colors represent brain regions with significant negative correlations. The color bar indicates the *t* value. L, left; R, right.

Multiple regression analysis revealed that PSQI scores in patients with CID were negatively correlated with GMV in several brain regions, including the right superior frontal gyrus-dorsolateral (SFGdor), bilateral superior frontal gyrus-medial (SFGmed), right gyrus rectus (REC), bilateral PreCG, right PoCG, left ITG, right MTG, and left supplementary motor area (SMA). The spatial distribution of these GMV regions exhibiting significant correlations is depicted in **Figure 3**. (The results used the same uncorrected threshold as above: voxel-level *p* < 0.001, cluster size ≥ 26). **Table 3** provides detailed information on these significant clusters. To quantify the effect sizes, we have added Cohen’s d to **Table 3**. Notably, converging evidence from the two independent analyses indicated that the bilateral PreCG exhibited both increased GMV and a negative correlation with PSQI scores.

**Table 3:** Multiple regression analysis revealed a significant negative correlation between gray matter volume and pittsburgh sleep quality index scores in several brain regions among participants with chronic insomnia disorder.

Brain regions	Anatomical classification	Whole cluster size	Cluster size	MNI coordinates			t score	Cohen’s d
				x	y	z		
R Superior frontal gyrus, dorsolateral	Prefontal	50	50	18	38	47	4.75	0.78
R Superior frontal gyrus, medial	Prefontal	35	31	5	39	57	4.96	0.79
L Superior frontal gyrus, medial	Prefontal	54	32	-6	32	32	3.97	0.69
R Gyrus rectus	Prefontal	43	30	33	56	-14	3.75	0.66
L Precental gyrus	Frontal	33	30	-51	0	42	3.68	0.67
R Precental gyrus	Frontal	96	60	38	-20	44	4.99	0.79
R Postcentral gyrus	Parietal		31	47	-18	53	3.57	0.71
L Inferior temporal gyrus	Temporal	56	56	-44	2	-36	4.61	0.78
R Middle temporal gyrus	Temporal	133	133	54	-47	6	3.76	0.87
L Supplementary motor area	Association	67	66	-6	6	69	3.54	0.67

Results were set at *p* < 0.001 (uncorrected, cluster ≥ 26 voxels). Abbreviations: MNI, Montreal Neurological Institute; L, left; R, right.

### 3.3 Global and regional network analysis

**Figure 4:**
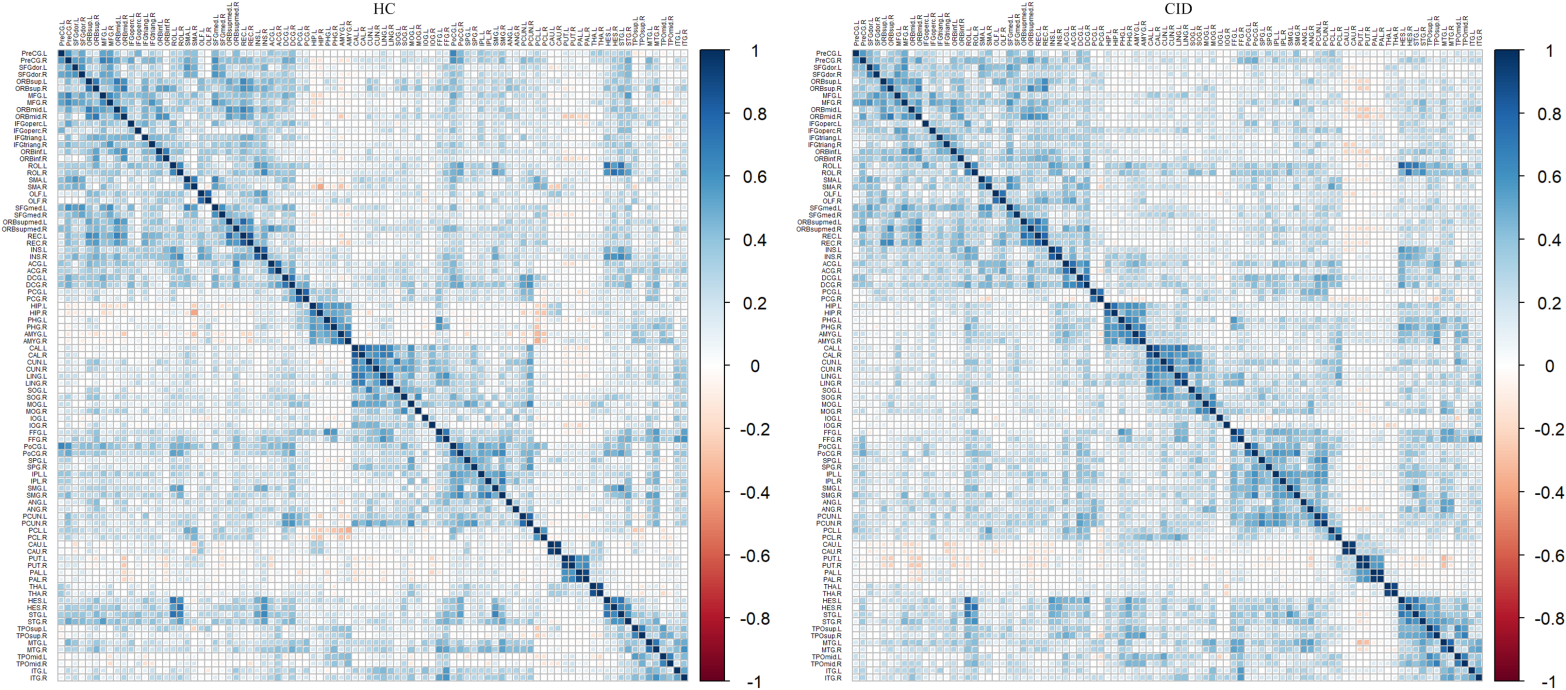
Association matrices between 90 regions of the automated anatomical labeling atlas for the HCs and CID groups. These matrices are the maps thresholded at the minimum network density (10%) in which the networks of both groups were not fragmented. The color bar shows the strength of the connections between any two nodes of the network. HCs, healthy controls; CID, chronic insomnia disorder.

**Figure 5:**
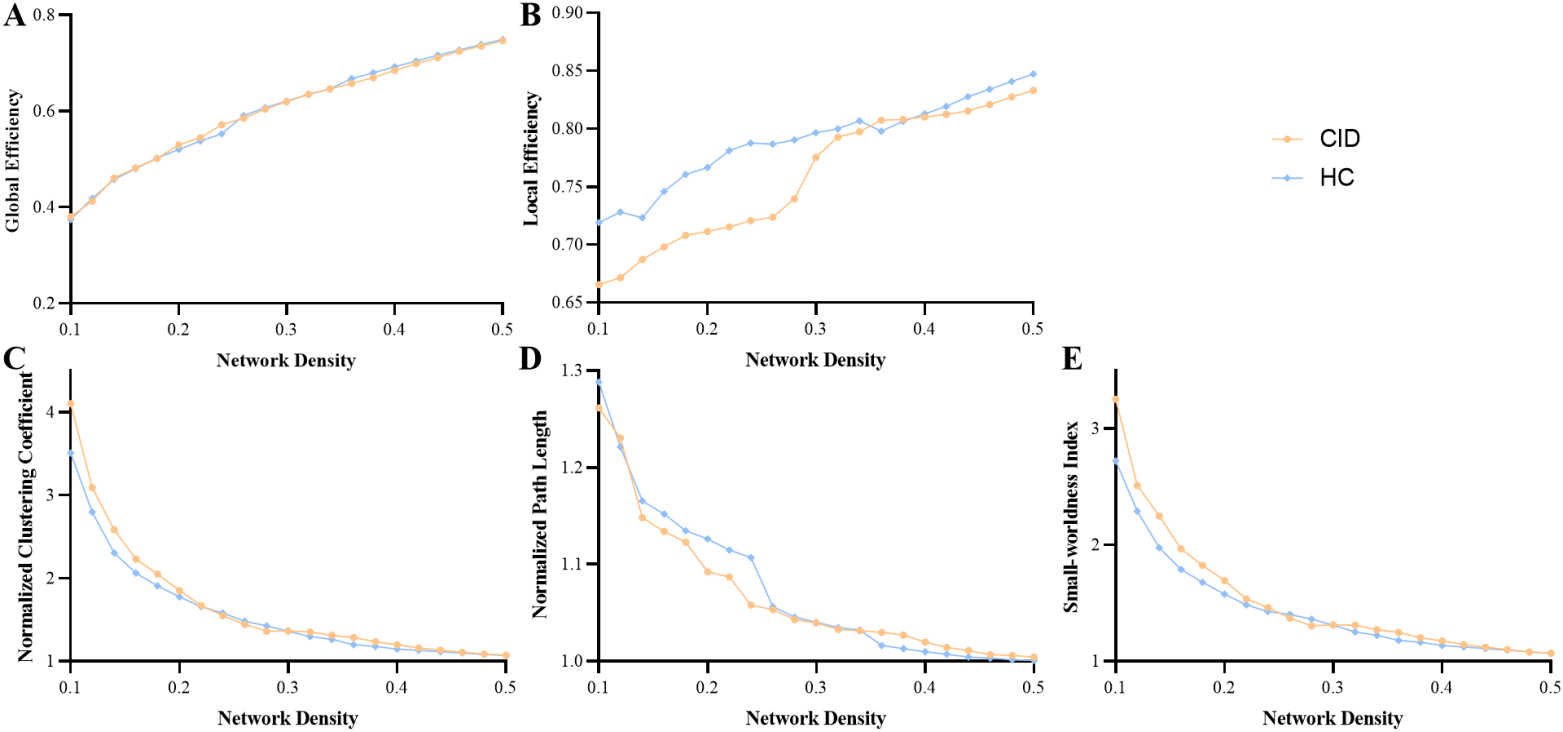
Changes in global network parameters as a function of network density in the CID and HC groups. **(A)** Global efficiency, **(B)** local efficiency, **(C)** normalized clustering coefficient, **(D)** normalized path length, **(E)** small-world index. HCs, healthy controls; CID, chronic insomnia disorder.

**Figure 6:**
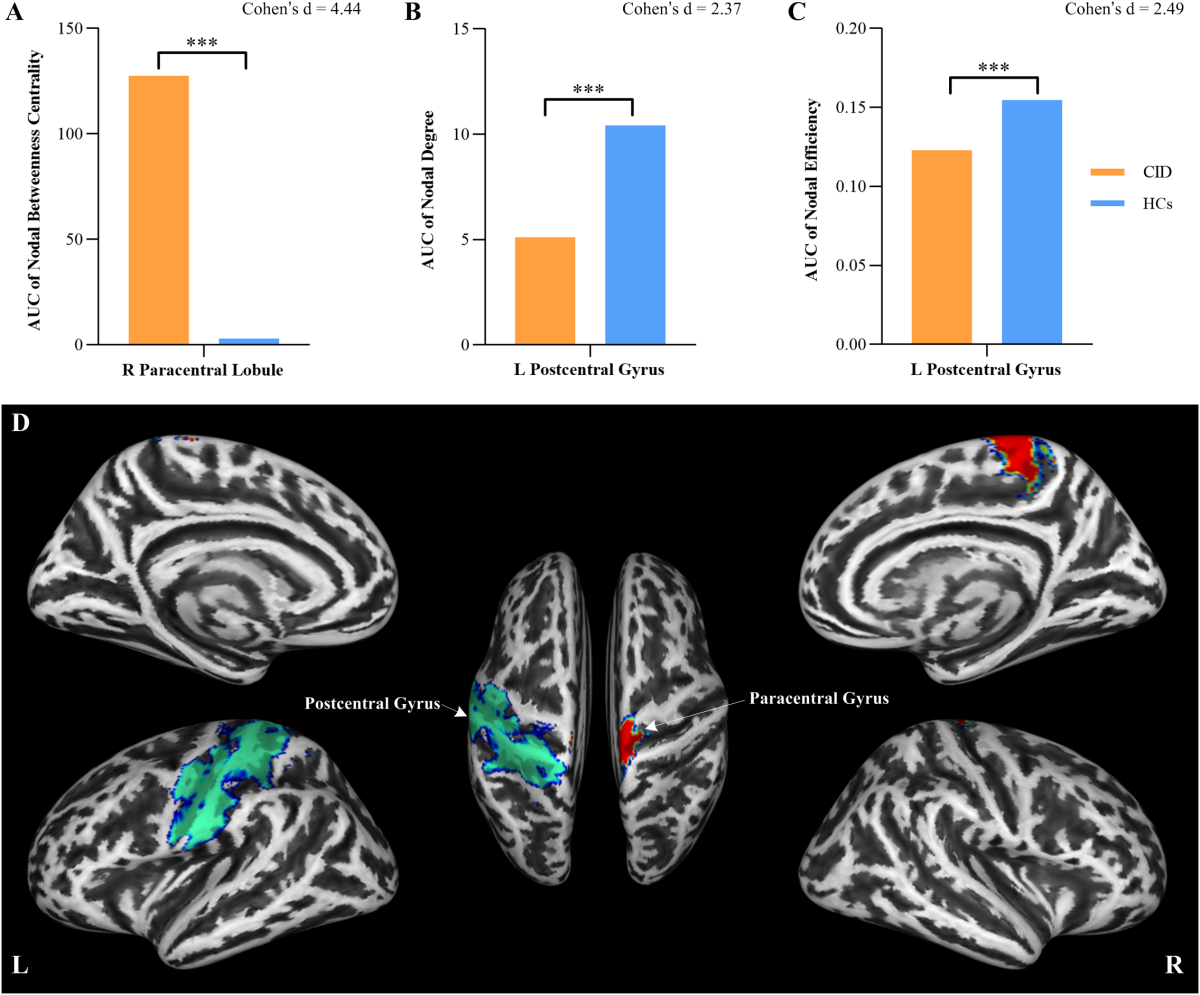
Regional network parameters differed between the HCs and CID groups. **(A)** The normalized nodal betweenness centrality of the right paracentral lobule. **(B)** The normalized nodal degree of the left postcentral gyrus. **(C)** The normalized nodal efficiency of the left postcentral gyrus. **(D)** Brain regions of interest: the left postcentral gyrus (green) and the right paracentral lobule (red). HCs, healthy controls; CID, chronic insomnia disorder, L, left; R, right. ***p < 0.001.

The correlation matrices revealed widespread positive correlations between most homotopic brain regions, as shown in **Figure 4**. The global network parameters across a range of network densities (sparsity thresholds from 0.1 to 0.5) are shown in **Figure 5**. The results indicate that both groups exhibited small-world topology across this range of densities, characterized by a normalized clustering coefficient greater than 1 (**Figure 5C**), a normalized characteristic path length approximately equal to 1 (**Figure 5D**), and consequently, a small-world index greater than 1 (**Figure 5E**). No significant differences were found in the AUCs of global topological properties between the two groups (all *p* > 0.05). However, compared with HCs, patients with CID showed a significantly increased AUC for nodal BC in the right paracentral lobule (PCL) (FDR-corrected *p* < 0.05; **Figure 6A**). Additionally, the CID group showed significantly decreased AUCs for both nodal degree and nodal efficiency in the left PoCG (FDR-corrected *p* < 0.05; **Figures 6B, C**). The spatial distribution of the left PoCG and the right PCL is depicted in **Figure 6D**.

### 3.4 Distributions of global hubs

**Figure 7:**
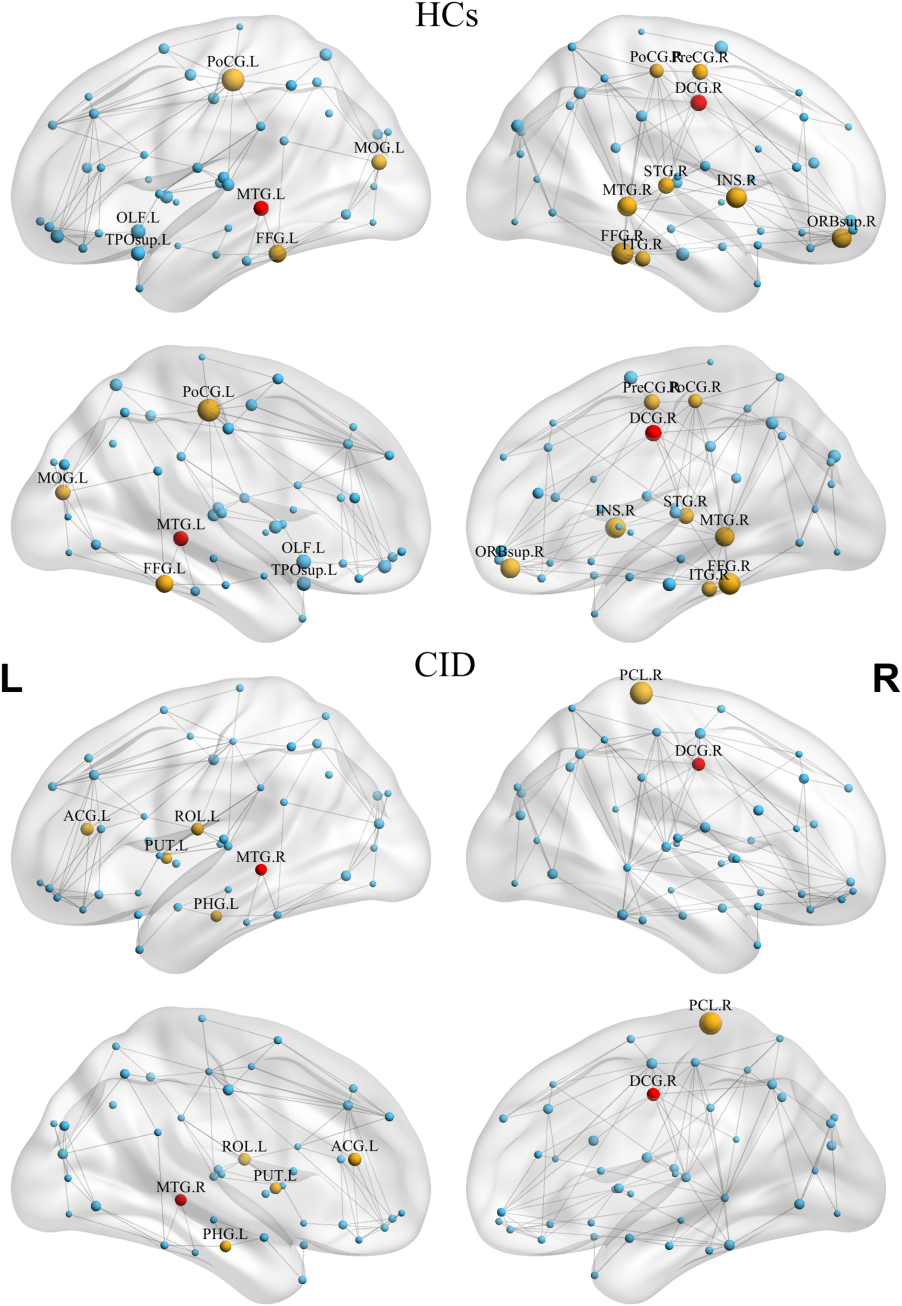
Distribution of Network Hubs in HCs and Patients with CID. The volume of each sphere represents the normalized nodal betweenness centrality of the corresponding brain region. Fifteen network hubs were identified in HCs, compared to seven in the CID group. Node colors denote group-specific hubs: yellow for hubs unique to each group, red for hubs common to both groups, and blue for non-hub regions. Detailed information on all network hubs is provided in **Supplementary Table S1**. HCs, healthy controls; CID, chronic insomnia disorder, L, left; R, right.

Furthermore, the distribution of global hubs across the seven canonical networks revealed 15 network hubs in patients with CID and seven in HCs (**Figure 7**). The right median cingulate/paracingulate gyri and the MTG were identified as hubs shared by both groups. Notably, the left PoCG and right PCL, which had shown altered nodal topological properties in the aforementioned analysis, were also identified as network hubs, each specific to either the CID or the HCs group. See **Supplementary Table S1** for details.

## 4 Discussion

Using VBM and graph theoretical analyses, we investigated GMV and the topological properties of the GMV-based SCN in patients with CID and HCs. This study yielded several key findings: First, the bilateral PreCG showed both increased GMV and a negative correlation with PSQI scores. Second, while no significant differences were found in the global network topological properties between groups, CID patients exhibited increased nodal BC in the right PCL and decreased nodal degree and efficiency in the left PoCG. Finally, we observed alterations in both the number and distribution of network hubs. Notably, the right PCL and the left PoCG that showed significant nodal parameter changes served as network hubs in either the CID or HC groups. Collectively, these findings are consistent with the hypothesis that the topological organization of the GMV-based SCN is altered at the nodal level in patients with CID.

### 4.1 GMV measures

Previous neuroimaging studies employing VBM have linked CID to GMV alterations in the frontal, temporal, and parietal cortices (7, 10). However, these findings remain inconsistent, highlighting the need for larger sample sizes and standardized analytical approaches to better characterize brain structural changes in CID (7–10). To address this issue, we enrolled a total of 82 CID patients and 73 HCs and utilized an advanced VBM algorithm to investigate GMV changes in patients with CID (33). To our knowledge, this study constitutes the largest sample to date investigating GMV alterations in patients with CID (5, 7). Our analysis revealed GMV increases in several regions, including the right insula, right TPOsup, left MTG, right STG, right FFG, right ANG, left IPG, left PoCG, bilateral PreCG, and right MOG. Notably, multiple regression analysis indicated that GMV in the bilateral PreCG was negatively correlated with PSQI scores. Although these findings are based on uncorrected statistical thresholds, they align with previous research conclusions regarding GMV hypertrophy in CID (8, 9). This consistency strengthens our confidence in the potential significance of this neural feature in CID. Therefore, these results provide preliminary supporting evidence for the theory, though rigorous validation in larger future cohorts remains imperative.

The PreCG, a key component of the sensory-motor network (SMN), is primarily involved in motor execution, sensorimotor integration, and working memory (42–45). Prior research has reported increased regional homogeneity (ReHo) in the bilateral PreCG, with ReHo values in the right PreCG negatively correlating with Self-Rating Depression Scale scores (42). Furthermore, William et al. observed enhanced functional connectivity between the primary sensory cortex and supplementary motor area among insomnia patients with difficulty falling asleep (46). The observed GMV increase in the PreCG and its negative correlation with PSQI scores may reflect heightened sensorimotor processing or hyperarousal, potentially contributing to difficulties in sleep initiation. Overall, this study reinforces the association between insomnia and neuroanatomical changes, proposing cortical hypertrophy as a potential morphological mechanism underlying the disorder.

### 4.2 Global network measures

The SCN has been suggested as a valuable tool for inferring large-scale structural brain networks (14, 23, 34). Previous studies have shown that the SCN corresponds to both functional networks and anatomical networks constructed through white matter tractography (25, 34, 47). This study revealed that both groups exhibited efficient and economic small-world topology across a range of densities. These results are consistent with previous graph analysis studies of CID patients, which consistently demonstrated a small-world architecture in functional networks and anatomical networks constructed through white matter tractography (25, 47). There were no significant differences in the topological properties of the global network between the two groups. These findings align with our previous study on brain functional networks in CID patients (25), suggesting that the segregation and integration of the global network are not significantly altered in patients with CID. However, patients with CID exhibited different topological properties of the global network (small-world, path length, clustering coefficient, local efficiency, and global efficiency) than HCs did (**Figure 5**). These altered global network properties indicate a disturbance in the network architecture of information transfer and processing across the brain in CID patients. Thus, further exploration of these differences using more sensitive methods is warranted in future studies.

### 4.3 Regional network measures

The regional network parameters, such as nodal BC, nodal degree, nodal efficiency, were compared between the two groups. Compared with HCs, patients with CID displayed an increased AUC of nodal BC in the right PCL. Additionally, there was a decreased AUC of the nodal degree and nodal efficiency in the left PoCG. Nodal BC refers to the fraction of all shortest paths in the network that pass through a given node and is used to identify important anatomical or functional connections (48, 49). Moreover, the nodal degree is defined as the number of connections a node has with the rest of the network and serves as a measure of the node’s interaction within the network (48, 49). Nodal efficiency measures the ability of a node to propagate information with the other nodes in a network (48, 49). These findings suggest that information transfer through the left PoCG is less efficient in CID patients than in HCs, whereas it is more efficient in the right PCL. The PoCG and PCL are located primarily in the SMN and are widely recognized for their involvement in sensory–motor processing, executive control, and emotion processing (50, 51). Functional MRI (fMRI) studies have previously shown significant correlations between PoCG and PCL activity and sleep quality (52). Furthermore, beyond the nodal parameter changes, this study also identified increased GMV in the insula, a key node of the salience network, which in the formation, expression, and perception of unpleasant emotions, processes central to the pathophysiology of CID (53–55). Previous studies have also identified altered connectivity patterns within and between the SMN and SN in relation to insomnia disorder symptoms (5, 52, 54). This study further supports these conclusions using GMV-based SCN and graph theory for the first time.

Enhanced morphometric similarity patterns in the right PCL were found to be associated with lower insomnia severity and fewer depressive symptoms in CID patients, suggesting a loss of distinctiveness within the SMN (51). In our previous study, we observed increased nodal centrality in the right PCL of brain functional networks (25). These findings suggest a potential association between structural covariance related to insomnia disorder and functional impairments. Dai et al. also reported increased neural hyperactivity in the right PCL following sleep deprivation (56). Increased functional connectivity (FC) between the right amygdala and PCL has been associated with mood disorders and suicidal behavior (50). Individuals with CID often experience a perpetuating cycle of somatic hyperarousal, heightened sensitivity to sensory stimulation, and increased cortical arousal, which in turn leads to difficulties in initiating and maintaining sleep (25). In conclusion, the observed increase in nodal BC in the right PCL may reflect a disruption in SMN-related function in CID symptoms within the SCN.

The PoCG is the main region responsible for processing external stimuli and is connected to the DMN, a functional brain network that shows synchronized activity even in the absence of external stimuli during wakeful rest and sleep (56). Compared with HCs, patients with migraine and insomnia presented changes in GMV in the PoCG, as well as decreased structural covariance integrity in the cerebellum (22). In patients with CID, resting-state fMRI studies have shown decreased regional homogeneity and amplitude of low-frequency fluctuations in the PoCG (57, 58). The bilateral PoCG was found to have decreased functional connectivity with the cuneus and superior frontal gyrus in CID patients, as reported by Dai et al. (59). Furthermore, a meta-analysis revealed reduced frontal–parietal activation following sleep deprivation (60). These findings collectively suggest that the decreased nodal degree and efficiency in the left PoCG in CID patients may disrupt integration and processing function of the SMN.

### 4.4 Network hubs

Based on nodal BC, patients with CID had fewer network hubs than HCs did (7 vs. 15) and exhibited a distinct spatial distribution of these hubs. While hubs in both groups were primarily located in frontal and temporal areas, consistent with previous reports (37), those in the CID group were predominantly located in brain regions with altered GMV. Notably, the left PoCG and right PCL, which showed altered nodal topological properties, were also identified as network hubs, with each specific to one of the two groups (the left PoCG in HCs and the right PCL in CID). This finding suggests that CID is associated with a reorganization of hub architecture within the large-scale SCN.

### 4.5 Limitations and strengths

The present study has several limitations. First, a key limitation of this study is the absence of objective sleep measurement using polysomnography (PSG), which remains the gold standard for the diagnosis and characterization of sleep disorders. Our reliance on subjective measures by PSQI instead of PSG may have introduced misclassification bias, potentially affecting the accurate differentiation of sleep disorder subtypes and severity. Consequently, the associations reported herein should be interpreted with caution, and future studies incorporating objective PSG data are warranted to confirm our findings. Second, our network construction has three main limitations: reliance on a specific parcellation scheme, exclusion of negative correlations, and a group-level approach. These issues limit the granularity and individual-level interpretability of the results. Consequently, future work should focus on developing more robust parcellation standards and new paradigms for individual-level network analysis (61). Third, this study did not employ diffusion tensor imaging (DTI) to construct a SCN for investigating abnormalities in the structural network of patients with CID. One key consideration stems from the work of Hidese et al., which revealed a significant negative correlation between PSQI-Japanese global scores and fractional anisotropy values across diffuse white matter regions (62). Additionally, network analysis derived from high-resolution diffusion-weighted imaging offers a more refined approach than group-level SCNs based on gray matter volume, as it allows direct assessment of the relationship between network metrics and clinical scores in individuals with CID (63). Finally, although we excluded patients with secondary insomnia due to depression or anxiety disorders, the well-established link between chronic insomnia and mood disturbances (64, 65) means we cannot definitively rule out the influence of residual symptoms. Therefore, we included HAMD and HAMA scores as covariates to control for these effects. Future work will recruit larger cohorts to better address generalization-related challenges.

### 4.6 Conclusion

In summary, this study revealed that while global network topology remained largely intact, CID patients exhibited regional alterations, including hypertrophic GMV and abnormal nodal properties within the GMV-based SCN. Specifically, the bilateral PreCG showed increased GMV, which negatively correlated with PSQI scores. At the nodal level, CID patients demonstrated increased nodal BC in the right PCL and decreased nodal degree and efficiency in the left PoCG. The constellation of regions exhibiting altered nodal parameters (the right PCL and left PoCG) also functioned as reconfigured network hubs. Collectively, our findings establishes an association between sleep disturbances in CID and aberrations in both the GMV of specific SMN nodes and their SCN topological properties, thereby providing new directions for elucidating the disorder’s pathophysiology.

## Data Availability

The raw data supporting the conclusions of this article will be made available by the authors, without undue reservation.
