# Supplementary material for: Disrupted gray matter structural covariance networks in chronic insomnia disorder
Source: Front Psychiatry. 2026 Jan 7;16:1629534. doi: 10.3389/fpsyt.2025.1629534 (PMC12819604; doi:10.3389/fpsyt.2025.1629534)
Supplement: Supplementary file 1 [file Table1.docx]

**Supplementary Materials**

**Table S1** Abbreviations and corresponding brain regions of network hubs in the healthy controls (HCs) and chronic insomnia disorder (CID) groups.

| **Groups** | **Hub Abbreviations** | **Hub Regions** |
| --- | --- | --- |
| **HCs** | PreCG.R | Right Precental gyrus |
|  | ORBsup.R | Right Superior frontal gyrus, orbital part |
|  | OLF.L | Left Olfactory cortex |
|  | INS.R | Right Insula |
|  | **DCG.R** | **Right Median cingulate and paracingulate gyri** |
|  | MOG.L | Left Middle occipital gyrus |
|  | FFG.L | Left Fusiform gyrus |
|  | FFG.R | Right Fusiform gyrus |
|  | PoCG.L | Left Postcentral gyrus |
|  | PoCG.R | Right Postcentral gyrus |
|  | STG.R | Right Superior temporal gyrus |
|  | TPOsup.L | Left Temporal pole: superior temporal gyrus |
|  | **MTG.L** | **Left Middle temporal gyrus** |
|  | MTG.R | Right Middle temporal gyrus |
|  | ITG.R | Right Inferior temporal gyrus |
| **CID** | ROL.L | Left Rolandic operculum |
|  | ACG.L | Left Anterior cingulate and paracingulate gyri |
|  | **DCG.R** | **Right Median cingulate and paracingulate gyri** |
|  | PHG.L | Left Parahippocampal gyrus |
|  | PCL.R | Right Paracentral lobule |
|  | PUT.L | Left Lenticular nucleus, putamen |
|  | **MTG.L** | **Left Middle temporal gyrus** |

The brain regions highlighted in bold represent overlapping nodes between the two groups, while the remaining regions correspond to hubs unique to each group.
